# Minimally Invasive Surgical Approaches to Colorectal Cancer: Learning Curve and Cost Considerations

**DOI:** 10.3390/cancers18193090

**Published:** 2026-09-23

**Authors:** Aniket Maini, Benjamin P. Gallant, Andrew Hu, Thomas Peponis

**Affiliations:** Division of Colon and Rectal Surgery, University of Massachusetts Memorial Medical Center, 67 Belmont St., Worcester, MA 01605, USA; aniket.maini@umassmed.edu (A.M.); benjamin.gallant@umassmed.edu (B.P.G.); andrewhumd@gmail.com (A.H.)

**Keywords:** cancer, colectomy, laparoscopy, proctectomy, robotics

## Abstract

Minimally invasive surgical (MIS) techniques have fundamentally transformed the management of colorectal cancer. Since the early 1990s, the field has rapidly evolved, shifting from initial concerns about oncologic safety to the widespread adoption of both laparoscopic and robotic surgeries. Specifically, laparoscopic approaches to colorectal cancer resection are now widely adopted and supported by robust randomized controlled trial evidence demonstrating favorable perioperative outcomes with equivalent oncologic results compared to open surgery. Robotic-assisted platforms have gained increasing adoption; however, their incremental clinical and oncologic benefit over laparoscopy remains under active investigation. As these technologies have been implemented, significant challenges related to surgical proficiency and economic sustainability have emerged. MIS approaches, particularly laparoscopic colectomies and proctectomies, involve steep, multidimensional learning curves that require substantial case volumes to achieve technical mastery and consistent clinical outcomes. Simultaneously, the rising costs of robotic platforms raise important considerations regarding the financial sustainability of healthcare systems. While robotic surgery may reduce certain downstream costs through shorter hospital stays and fewer complications, its substantially higher upfront operative costs and uncertain cost-effectiveness relative to laparoscopy require careful consideration, and access remains a barrier, particularly for patients without private insurance. This narrative review describes the evolution of MIS in colorectal cancer treatment and examines the dual considerations of the surgical learning curve and hospital cost implications.

## Simple Summary

Minimally invasive surgery (MIS) for colorectal cancer has improved patient outcomes but presents significant challenges related to surgeon training and hospital costs. This review examines the learning curves associated with laparoscopic and robotic techniques for managing colon and rectal cancer. It also analyzes the financial implications of these technologies on healthcare systems. This narrative review demonstrates that MIS is associated with favorable perioperative outcomes while maintaining appropriate oncologic outcomes in selected patients; institutional support and cost-reduction strategies are essential to make these surgeries accessible to all patients.

## 1. Introduction

The advent of minimally invasive surgical (MIS) techniques in colon and rectal surgery has fundamentally transformed patient care, ushering in an era of enhanced surgical precision, reduced patient morbidity, and improved postoperative outcomes [1,2]. The transition from traditional open procedures to MIS for a wide range of colorectal pathologies, including contemporary oncologic resections, represents a paradigm shift in perioperative care.

Although both laparoscopic and robotic techniques have shown significant potential, the practical implementation of these systems has been a subject of ongoing discussion. With rising healthcare costs, hospital overhead, and high upfront device prices, acquiring and maintaining such systems requires substantial investment from healthcare institutions. Additionally, there is a considerable learning curve for residents and surgeons striving to attain proficiency in minimally invasive techniques, necessitating a combination of training, operative experience, case volume, and case diversity. Addressing both the financial burden and the learning curve is a critical challenge to effectively balance institutional capabilities with the delivery of high-quality care. This narrative review aims to fill this gap by being among the first to integrate these two key considerations, examining both the cost implications and the learning curve associated with MIS techniques in colorectal cancer management.

### Search Methods

A literature search was conducted across PubMed, Scopus, and Google Scholar to identify relevant articles published through 25 July 2026. For PubMed and Scopus, search strings included the following: (“laparoscopic” OR “robotic” OR “minimally invasive”) AND (“colectomy” OR “proctectomy” OR “colon” OR “rectal” OR “colorectal surgery”) AND (“cancer” OR “adenocarcinoma”) AND (“learning curve” OR “operative time” OR “proficiency”) for learning curve-related articles, and (“laparoscopic” OR “robotic” OR “minimally invasive”) AND (“colectomy” OR “proctectomy” OR “colon” OR “rectal” OR “colorectal surgery”) AND (“cost” OR “economic” OR “financial”) for cost-related articles. The searches covered all articles published through 25 July 2026. Google Scholar searches were conducted using similar term combinations; given the large volume of results generated by this platform, results were reviewed until diminishing relevance was observed, typically within the first 10 to 15 pages of results, and articles were selected based on their direct relevance to the learning curve or cost considerations of minimally invasive surgery for colorectal cancer. We identified additional studies through manual reference checks of included articles.

The initial searches identified a range of 712 to 2733 learning curve-related articles and 1054 to 2107 cost-related articles across PubMed and Scopus, respectively. Titles and abstracts were screened by two independent reviewers (A.M. and B.G.), yielding approximately 250 total articles for full-text review. Of these, 43 articles were ultimately included based on their relevance to the studied topic. Peer-reviewed original research articles, systematic reviews, and meta-analyses published in English were considered for inclusion. Conference abstracts, editorials, and non-peer-reviewed publications were excluded, as were studies focusing exclusively on non-oncologic colorectal indications. Disagreements regarding study inclusion were resolved through discussion and consensus with a senior author (T.P.).

## 2. Learning Curve Considerations

The surgical learning curve is inherently multifactorial and has been defined using heterogeneous endpoints. The surgical learning curve is used as a metric to determine whether a trainee has achieved proficiency after a certain number of cases and is valuable for assessing trainee progress [3]. Early studies and definitions of the surgical learning curve for laparoscopic and robotic-assisted laparoscopic colorectal cancer procedures established learning curve case volumes based on single parameters and averaging outcomes such as operative time, complication rates, blood loss, hospital length of stay, morbidity and mortality, and conversion rates. However, some studies employed a CUSUM (cumulative sum) analysis, which provides a more credible and nuanced multidimensional assessment of operative proficiency [4]. CUSUM analyses are effective for sequentially tracking performance over time by sequentially plotting running totals of deviations of observed outcomes from predefined targets and, in this context, can help identify when a surgeon’s performance improves and subsequently stabilizes.

Additionally, it is important to recognize the variability in statistical methods used to analyze learning curves beyond traditional CUSUM analysis. For example, risk-adjusted CUSUM analyses account for patient- and case-level risk factors, allowing for performance to be evaluated relative to expected outcomes based on case complexity rather than as absolute deviations [5]. Moving average analyses are also employed; moving average analyses smooth performance trends over time by averaging outcomes across a rolling window of consecutive cases, reducing the influence of individual outlier cases and providing a clearer picture of overall trajectory [6]. Operative time learning curves are often used as surrogates for proficiency by tracking procedure durations and are commonly applied in studies of this nature [7]. Composite surgical-failure endpoints can be analyzed to establish undesirable outcomes as benchmarks for “failure” if any such outcomes occur; these are frequently used in surgical oncology to assess proficiency [8]. Finally, oncologic and postoperative outcome-based proficiency endpoints are commonly utilized to evaluate actual patient outcomes as proxies for proficiency, rather than relying solely on intraoperative measures. Given this heterogeneity in analytical approaches—and recognizing that the resulting case-volume thresholds are highly dependent on the specific endpoint and method chosen—a more detailed examination of these individual domains within colorectal procedures will better elucidate the determinants of surgical proficiency.

### 2.1. Laparoscopic Colectomies

As mentioned, investigating the laparoscopic learning curve for colonic resections demands a multidimensional analysis, preferably involving a large case series. A large study published in 2005 from the Cleveland Clinic analyzed clinical data from 900 patients undergoing laparoscopic colorectal procedures and determined learning curves using risk-adjusted CUSUM analyses. Key metrics examined in the study included conversion rates (8.1% for right-sided resections and 15.3% for left-sided resections), readmission rates (10.5% for right-sided resections and 6.2% for left-sided resections), postoperative complication rates (20.8% for right-sided resections and 17.8% for left-sided resections), and operative time, which showed a statistically significant decrease as operative experience increased. Based on these indices, the CUSUM analysis identified a learning curve of 55 cases for right-sided colectomies and 62 cases for left-sided colectomies [9].

A systematic review of the literature involving 4852 cases of laparoscopic colorectal procedures analyzed learning curves and case proficiency rates based on specific operative metrics, thereby defining more refined benchmarks. Although the study broadly included “laparoscopic colorectal surgery” and did not extrapolate by procedure type, the study identified the learning curve plateaus reported by surgeons for conversion rate at 152 cases, complications at 143 cases, operative time at 96 cases, blood loss at 87 cases, and hospital stay at 103 cases [10]. A similar study analyzed specific operative metrics in laparoscopic colorectal resections to determine learning curves, including operative time for right hemicolectomies and anterior resections, both of which continuously decreased over time. This study also demonstrated a decline in anastomotic leak rates after 200 cases and stabilization in the number of harvested lymph nodes in right hemicolectomies after 200 cases [11].

A recent learning curve analysis examined prospective data from 2015 to 2021 involving 202 patients who underwent laparoscopic complete mesocolic excision (CME) for right-sided colon cancer [12]. This study is particularly noteworthy because the learning curve for CME in colon cancer reflects the procedure’s technical complexity and the potential for significant vascular complications involving the superior mesenteric vein, among other technical challenges. Nevertheless, CUSUM and risk-adjusted CUSUM analyses identified proficiency in this procedure as being achieved between 24 and 33 cases. The study also divided the learning curve into two phases: an initial phase focused on overcoming the learning period by acquiring technical competence, and a subsequent proficiency phase characterized by substantial reductions in the surgical failure rate [12].

### 2.2. Robotic Colectomies

Robotic surgery has introduced numerous advances in perioperative care but also presents unique challenges for trainees as the literature on the learning curve of robotic surgery remains relatively limited. A recent report attempted to outline the learning curve in robotic colorectal surgery, highlighting classic models with many of the previously mentioned metrics, but also identifying characteristics unique to the robotic system. These include metrics such as docking time and console and operative times, along with conversion rates and postoperative outcomes. This study further emphasized that learning curves in robotic colorectal surgery may be heavily influenced by extraneous factors due to the novel nature of the technology, including prior overall experience in colorectal surgery, experience in the robotic surgical unit, case variability, robotic simulation, and time spent under surgical mentorship [13].

There is substantial variability in reported learning curves for specific oncologic resections of the colon. A large series involving 502 patients between 2009 and 2018 analyzed a range of colorectal procedures, including right and left hemicolectomies and sigmoidectomies, and found significant differences in learning curves among these surgeries [14]. For right hemicolectomies, no clear learning curve was established for procedural proficiency, and there was no significant decrease in operative times between the first and fourth quartiles of cases. In contrast, left hemicolectomies and sigmoidectomies demonstrated a learning curve, with proficiency achieved after 35 to 45 cases, with a significant reduction in operative times from the first to the fourth quartile of cases [14]. However, learning curves in this study were not extrapolated by individual metrics such as complication rate, operative times, blood loss, etc.

A 2015 study analyzed the surgical learning curve for 108 oncologic robotic right hemicolectomies with intracorporeal ileocolic anastomoses—relatively complex procedures that require practice among trainees [15]. This prospective analysis employed CUSUM and risk-adjusted CUSUM methods and determined that the learning curve for robotic right hemicolectomies with intracorporeal anastomoses peaked at approximately 44 cases. Graphical extrapolation from this study identified three distinct phases: the initial learning phase (cases 1–44), a second consolidation period (cases 45–90), and a third mastery phase (cases 91–108). Based on these findings, the investigators determined that proficiency in operative time, conversion rates, and lymph node harvest was achieved after 44 cases, with continued improvements observed across the three phases [15].

Further investigation into the learning curve for robotic colectomies using large datasets would be valuable in clarifying how these curves differ between specific procedures within this space. For example, de’Angelis et al. compared learning curve data for a single surgical fellow’s 30 patients undergoing robotic right colectomy and 50 patients undergoing laparoscopic right colectomy [16]. Their data showed a learning curve of 16 cases for robotic colectomies and 25 cases for laparoscopic colectomies. Additional findings from this study indicated no significant differences in operative time between the two techniques; however, operative time significantly decreased for both as the number of cases increased [16]. Overall, further data comparing learning curves for laparoscopic and robotic colectomies would be valuable for academic institutions to optimize resident and fellow training programs.

### 2.3. Laparoscopic Proctectomies

The previous literature has highlighted the significant inherent challenges associated with laparoscopic proctectomies and oncologic rectal resections, primarily due to the anatomical complexity of the deep pelvis. Therefore, establishing reliable learning curve data for total mesorectal excision (TME) is essential for trainees to monitor their laparoscopic progress, enhance their technical proficiency, and ensure consistent clinical and oncologic outcomes. A substantial body of literature discusses learning curves for various rectal procedures, considering both overall case volumes and learning curves stratified by operative metrics, as mentioned previously. Burghgraef et al.’s systematic review, published in 2022, analyzed learning curves from 45 studies with an average of 205 laparoscopic TME (L-TME) patients, and determined that the risk-adjusted CUSUM analysis indicated a learning curve of 50–70 procedures for this technique [17]. However, this study also noted significant variability in the data, largely attributed to factors such as surgeon experience and patient characteristics.

Son et al. conducted a retrospective analysis of 431 patients from 1994 to 2006 who underwent laparoscopic proctectomy for rectal cancer by a single surgeon. These laparoscopic procedures included anterior resections, low anterior resections, laparoscopic abdominal transanal proctosigmoidectomy with coloanal anastomosis (LATA), abdominoperineal resections, and Hartmann’s resections. Using risk-adjusted CUSUM analyses, they reported an effective learning curve between 60 and 80 procedures. Other metrics, such as conversion rate (61 cases), complication rates (79 cases), and operative time (61–75 cases), showed continuous improvement throughout the series [18]. Park et al. observed a steady decline in operative time after approximately 90 cases [11]. Similarly, Bege et al. studied 127 patients undergoing laparoscopic conservative mesorectal excisions between 2002 and 2006. They found that a success rate based on oncologic data and procedure outcome peaked around 50 cases, then decreased thereafter. Operative time in this study gradually decreased over time, consistent with other findings. Although no learning curve or peak was identified for conversion rates, postoperative morbidity peaked around 45 cases [19]. Kayano et al. further supported these findings in a study of 250 patients undergoing laparoscopic lower anterior resections over 14 years. Their moving average analysis indicated stabilization after 50 cases, with conversion rates decreasing between 151 and 200 cases and postoperative complications declining between 201 and 250 cases [20]. Additionally, learning curves for recurrence rates were reported in a series of 317 patients undergoing laparoscopic lower anterior resection using risk-adjusted CUSUM analyses. They determined that 110 cases were necessary to reduce locoregional recurrence, while 50 to 70 cases were required to minimize circumferential resection margin involvement [21].

Landmark clinical trials are frequently discussed in this context due to their significant role in establishing national and international guidelines for laparoscopic rectal surgery. For example, the Comparison of Open versus laparoscopic surgery for mid or low REctal cancer After Neoadjuvant chemoradiotherapy (COREAN) trial, conducted from 2006 to 2009, investigated survival outcomes comparing laparoscopic and open approaches. Surgeons participating in this trial were required to have completed at least 91 laparoscopic colorectal cancer excisions, with total mesorectal excisions (TMEs) recommended to be between 50 and 80 procedures [22]. Likewise, the Laparoscopy-assisted versus open surgery for low rectal cancer (LASRE) trial analyzed three-year survival outcomes for 1070 patients from 2013 to 2018 and required surgeons to have completed a minimum of 100 laparoscopic TME procedures [23]. It should be noted that surgeon experience requirements in clinical trials represent credentialing criteria designed to minimize learning-curve effects on trial outcomes and should not be interpreted as empirically validated thresholds for proficiency in routine clinical practice.

### 2.4. Robotic Proctectomies

With regard to proctectomies performed using robotic techniques, Burghgraef’s systematic review identified a learning curve for robotic-assisted TME ranging from 32 to 75 procedures, with an operative time learning curve spanning 12 to 35 cases. This study also importantly highlights how the learning curves for robotic TME (R-TME) are specifically influenced by a surgeon’s experience with L-TME, as privileges with the robotic system are generally preceded by completion of laparoscopic cases during training [17].

Similarly to the existing literature on robotic colectomies, various single-surgeon series utilizing robotic surgery for rectal cancer resections have been reported to determine learning curves. A single-surgeon series of 167 patients undergoing robotic TME between 2007 and 2012 in Korea employed a risk-adjusted CUSUM analysis to identify the composite learning curve peak, observed at approximately 32 cases [24]. The study also reported the learning curve for operative time, where a moving average revealed two individual peaks at 33 and 72 cases. Consequently, the learning process here was divided into three phases: cases 1–32, cases 33–72, and the subsequent 95 cases, during which a steady decrease in operative time was observed [24]. A similar study by Tang et al. examined 389 consecutive patients undergoing robotic TME between 2015 and 2018. This single-surgeon series also used a risk-adjusted CUSUM analysis to determine a learning curve peak near that of the previous study at 36 cases and divided the phases into learning (cases 1–36) and mastery (cases 37–389) [25]. They also found that operative time steadily decreased, particularly during the mastery phase. However, variability exists among these reports; a propensity score-matched study analyzing learning phases of a single-surgeon series of 506 patients undergoing robotic TME for rectal adenocarcinoma between 2007 and 2018 utilized risk-adjusted CUSUM analyses for surgical failure (including conversion to open surgery, severe complications, etc.) and found the learning phases to be considerably longer, with phase 1 spanning cases 1–177 and phase 2 cases 178–506 [26]. This suggests that larger case series may provide more comprehensive data to more accurately define these learning curves. Reports of specific operative metrics for the robotic approach remain relatively scarce compared to the laparoscopic technique. However, a 2023 multicenter French study of 174 patients undergoing robotic rectal cancer resections identified a learning curve of 12 to 21 cases for operative time and 9 to 14 cases for conversion rate [27]. This study is also notable for demonstrating a significant increase in lymph node harvest after the initial learning phase of 12 to 21 cases.

Similarly to laparoscopic proctectomies, landmark trials have established specific requirements for surgeons performing robotic rectal cancer resections. For example, the ROLARR randomized clinical trial, which compared conversion rates in rectal cancer patients undergoing robotic-assisted versus traditional laparoscopic surgery, required surgeons to have completed at least 30 minimally invasive rectal cancer resections, including a minimum of 10 robotic-assisted laparoscopic procedures [28]. Similarly, the previously mentioned REAL trial mandated that surgeons complete at least 50 robotic rectal cancer resections and submit robotic TME video footage for assessment of surgical proficiency before participating in the trial [29]. A summary of the learning curve considerations for laparoscopic and robotic colectomies and proctectomies is displayed in Table 1. Overall, these data further underscore the importance of understanding learning curve metrics in this field. Greater precision in defining distinct learning curve peaks and plateaus will enable surgeons to better identify performance trends, optimize training for faculty and residents, and sustain long-term improvement.

It is critical to highlight the evident variation in reported learning curves among the sources cited in this review. Factors such as heterogeneity in study design, definitions of proficiency, patient volume, and individual surgeon operative experience may substantially impact when proficiency is achieved and recorded. As shown in Table 1, some single-surgeon series demonstrated learning curves that were substantially lower than those reported by retrospective or prospective cohort studies for the same class of procedures, illustrating that individual surgeon experience likely plays a key role in case numbers required to achieve proficiency. However, among single-surgeon series themselves, overall patient volume did not appear to have a major impact on reported proficiency thresholds. For instance, Bege reported an overall learning curve of 50 cases within a cohort of 127 cases, whereas Son reported a peak at 60–80 cases in a cohort of 431 patients [18,19]. Further investigation and explicit data on how surgeon volume influences learning curves would benefit the surgical community and aid institutions, particularly in determining case distribution for trainees.

Given this variability, not all reported learning curve thresholds are equally suited to inform training standards. Single-parameter estimates—such as operative time alone—are the most commonly reported but are also the most susceptible to confounding by case mix, patient complexity, and institutional factors, and should be interpreted cautiously as standalone benchmarks. In contrast, multidimensional analyses employing risk-adjusted CUSUM methods that simultaneously assess operative time, conversion rate, complication rate, and oncologic adequacy of resection provide the most robust and clinically meaningful proficiency thresholds. Among the studies reviewed, the most generalizable estimates for training purposes are those derived from large prospective or multicenter cohort studies using composite endpoints: approximately 55–62 cases for laparoscopic colectomy, 50–70 cases for laparoscopic TME, and 32–44 cases for robotic TME. These figures align closely with the surgeon experience requirements of major randomized trials and represent the best available approximation of proficiency benchmarks, while recognizing that individual surgeon trajectories will vary and that no single threshold can substitute for ongoing competency-based assessment.

## 3. Cost Considerations

As laparoscopic and robotic approaches to the surgical treatment of colon and rectal cancer have gained popularity, the associated costs have also increased. To date, multiple studies have demonstrated the advantages of these approaches, including reduced intraoperative blood loss, decreased postoperative pain, and a shorter hospital stay. These factors play a significant role in mitigating the costs associated with minimally invasive surgery. Furthermore, as access to these surgical approaches improves, future costs are expected to decrease even further. This section will examine the costs associated with colectomies and proctectomies for colon and rectal cancer management, as well as their implications for patients and healthcare institutions. It is important to note that direct comparisons of absolute monetary values across the economic studies listed here should be made with caution. The economic literature summarized in this section spans multiple countries, healthcare systems, time periods, and currencies, and the studies vary in perspective (payer or hospital), costing methodology, inflation adjustments, cost-components included (some capture only index hospitalization costs while others incorporate readmissions, post-discharge care, and long-term expenditures), and time horizon. As a result, the cost differences described are best understood as directional estimates rather than precise cross-study benchmarks.

### 3.1. Laparoscopic Colectomies

Laparoscopic colectomies have demonstrated oncologic outcomes comparable to those of open surgery, while also being associated with significant cost reductions for U.S. payers compared to open colectomies. One study analyzed over 7000 patients using the IBM MarketScan Database, a payer-perspective claims analysis with costs captured from the index surgery through one year postoperatively, and found that minimally invasive colectomies had lower mean expenditures than open surgery at both the index surgery (costs were $34,588 (MIS) vs. $36,975 (open), *p* < 0.001) and up to one year postoperatively ($20,051 (MIS) vs. $24,309 (open), *p* < 0.001) for those who did not undergo adjuvant chemotherapy [30]. These savings also translated to those patients who did receive adjuvant chemotherapy. Another study from 2017, using the MarketScan Commercial Claims database, examined 1266 laparoscopic and open colectomies for colon cancer in the U.S. from 2012 to 2013 and demonstrated that laparoscopic colectomies had lower risk-adjusted combined inpatient and 30-day costs compared to open approaches ($36,395 for laparoscopic vs. $44,226 for open colectomy, *p* < 0.001) [31]. Additionally, this study reported lower readmission rates and associated readmission costs ($1676 vs. $3151, *p* = 0.031) in the laparoscopic cohort compared to open colectomies. These findings are corroborated by other databases as well. A 2019 study analyzing 531,536 patients undergoing surgical treatment for colon cancer between 2008 and 2014 using the Nationwide Inpatient Sample database found propensity-matched median costs of $14,487 for laparoscopic vs. $18,541 for open colectomy [32]. Laparoscopic surgery was also associated with shorter hospital length of stay and lower mortality compared to open colectomies.

While these studies demonstrate that overall direct costs favor laparoscopic surgery, a smaller case-matched analysis from 2003 showed higher upfront operating room costs for laparoscopic procedures [33]. The cohorts in this case-matched study were matched based on disease-related factors, and the authors found that direct operating room costs were significantly higher after laparoscopic surgery (*p* < 0.001). However, the hospital length of stay was significantly shorter, contributing to lower total costs (*p* < 0.001) for the laparoscopic group [33]. The authors attributed the reduced total cost to lower pharmacy, laboratory, and ward nursing expenses. It should be noted, however, that this study was conducted over two decades ago, during the early adoption of laparoscopic surgery, which may have contributed to the increased initial operating room costs.

### 3.2. Robotic Colectomies

Currently, the American Society of Colon and Rectal Surgeons (ASCRS) and the Society of American Gastrointestinal and Endoscopic Surgeons (SAGES) give a strong recommendation (1A) for a minimally invasive surgical approach when expertise is available. This recommendation is supported by high-quality randomized controlled trial (RCT) evidence from the ALCCaS and COLOR trials [34]. Upfront operative costs of minimally invasive surgical techniques are offset by lower overall costs due to shorter hospital stays, reduced intensive care utilization, fewer complications, and lower readmission rates [33,35]. Similar findings have been reported for robotic colectomies compared to open procedures, although robotic surgery incurs higher upfront operative costs, exceeding those of both laparoscopic and open colectomies’ direct costs ($15,133 for robotic vs. $10,714 for laparoscopic vs. $12,849 for open), according to a 2022 analysis of 3432 colectomies [36].

When directly comparing laparoscopic and robotic colectomies, Chiu et al. found that mortality rates and general medical complications were equivalent between the two groups, but the median cost was lower in the laparoscopic group ($14,641 vs. $16,628) [32]. Similar results were observed for both right and left colectomies. One study reported that the overall cost increase for robotic right colectomy was 20% ($12,516 vs. $15,027) and left colectomy was 24% ($14,157 vs. $17,493) compared to laparoscopy [37]. However, it should be noted that this study did not focus exclusively on laparoscopic and robotic colon cancer resections.

While the superiority of robotic surgical techniques over laparoscopic approaches regarding oncologic outcomes has not yet been fully established, the cost remains significantly higher. A landmark 2020 study by Simianu et al. found that robotic colectomy costs $745–$1339 more per case than its laparoscopic counterpart [38]. Although this study accounted for all indications for colectomy, the authors further demonstrated an absence of meaningful differences in quality-adjusted life years (QALYs) between the two cohorts. Specifically, the incremental cost-effectiveness ratio (ICER) exceeded $2 million per QALY, a figure driven by the negligible quality of life differences between modalities [38]. These findings underscore a critical need to minimize instrument costs, reduce waste, and improve postoperative outcomes to offset the inherent financial burden of robotic surgery.

### 3.3. Laparoscopic Proctectomies

Laparoscopic proctectomies for rectal cancer share similarities with laparoscopic colectomies, as their total costs are often offset by fewer complications and shorter hospital length of stay compared to open resections. However, on average, they incur significantly higher total 90-day hospital costs [35]. The Australasian Laparoscopic Cancer of the Rectum Trial (ALaCaRT) included a secondary endpoint analysis of 468 randomized patients and found that the laparoscopic procedure cost $521 Australian dollars (AUD$) more than the open procedure (AUD$4542 vs. AUD$4021, *p* < 0.001), primarily due to longer operative times and more expensive equipment [39]. However, at 12 months, the total healthcare costs were slightly lower for the laparoscopic group, although the difference was not statistically significant (AUD$43,288 laparoscopic vs. AUD$45,384 open; 95% CI: −AUD$9358 to AUD$5003). The authors attributed these equivalent costs at one year to shorter subsequent hospital stays.

Further studies have validated the cost-effectiveness of laparoscopic proctectomy compared to the open approach. In 2014, Keller et al. demonstrated these findings through a case-matched analysis of 254 patients with rectal cancer [40]. The cost model in this case-matched analysis incorporated operating-room time, post-discharge ICU, skilled-nursing, and home-care costs. The authors found that laparoscopic proctectomy resulted in significantly lower total hospital costs (*p* < 0.01) and a shorter hospital length of stay (*p* < 0.01) than open surgery [40]. Additionally, both operative time (*p* < 0.01) and cost per operating room minute (*p* = 0.04) were higher in the open group; furthermore, significantly more patients in the open group required ICU care (*p* = 0.03), skilled nursing (*p* = 0.03), or home care services (*p* < 0.01) upon discharge. Ultimately, these data suggest that, like laparoscopic colectomy, the laparoscopic approach to rectal cancer resection offers a superior economic profile by reducing both perioperative resource utilization and post-discharge care requirements.

### 3.4. Robotic Proctectomies

Historically, robotic platforms have consistently incurred higher costs than laparoscopic approaches. Moreover, studies have shown that patients with higher incomes and private insurance are more likely to undergo robotic colorectal cancer surgery than those with public insurance (i.e., Medicaid or Medicare) or no insurance [41,42,43,44]. Several studies have shown that robotic proctectomy incurs higher costs compared to laparoscopic and open approaches. A single-institution study from 2017 compared the costs for primary rectal cancer patients undergoing curative-intent proctectomy. The study found no significant difference in short-term oncologic outcomes; however, the direct hospitalization costs for the primary resection and the total direct costs (including readmissions and ileostomy closure hospitalizations) were significantly higher in the robotic cohort [45]. The authors emphasized the importance of reducing the cost of robotic procedures if robotic surgery is to be widely adopted. More recent literature supports the findings of this 2017 study. A 2024 study by Gebhardt et al. retrospectively examined laparoscopic and robotic rectal cancer resections from 2016 to 2021 at a single German institution, in a hospital-perspective analysis reporting inpatient costs in euros without inflation adjustment. The reported median total inpatient costs for robotic approaches were higher than those for laparoscopic surgeries (€17,663 vs. €14,089, *p* = 0.018), with upfront surgery costs also greater for the robotic approach (€10,156 vs. €7468, *p* < 0.0001) [46]. Examples of the cost considerations for laparoscopic and robotic colectomies and proctectomies are demonstrated in Figure 1. As the adoption of robotic surgery continues to rise, addressing these financial barriers is essential to ensure that robotic colorectal surgery becomes both affordable and accessible to all patients regardless of socioeconomic status.

### 3.5. Emerging Robotic Platforms

Although the da Vinci robotic system has been the most commonly used robotic platform for colorectal surgery in the United States, emerging surgical technologies are likely to greatly alter financial considerations with regard to robotic colorectal surgery by increasing market competition and lowering hospital acquisition costs. Hussain et al. investigated how the Versius, Senhance, and Revo-I systems are slowly being introduced into colorectal surgery due to their potential to reduce costs, yet they currently lack major colorectal trials to suggest they may replace the da Vinci system [47]. Although these new platforms have been primarily studied in urological surgery, they still show promise and may contribute to modifying the financial implications of system acquisition while improving surgical augmentation through haptic feedback and ergonomics [48,49]. Medtronic’s Hugo Robotic Assisted Surgery (RAS) system has also emerged in da Vinci’s space and has undergone preliminary clinical implementation in urologic and gynecologic surgery [50,51].

It is critical to acknowledge the limited generalizability of the financial implications and economic comparisons mentioned in this review. Fiscal differences exist across healthcare systems in different nations, driven by variable reimbursement models, distinct currencies, and varying hospital ownership structures globally, all of which impact the applicability of reported cost estimates. Additionally, individual hospital-based metrics and expenses influence this cost discussion, as surgeon and institutional volume, alongside the upfront and maintenance costs of robotic systems, are significant.

### 3.6. Limitations

This narrative review is subject to several inherent methodological limitations. There is the potential for selection bias given the methodology used for study selection and the exclusion of studies in languages other than English. In addition, no formal quality appraisal tools were applied to the included studies, which vary considerably in design, sample size, and analytic methodology, ranging from single-surgeon retrospective series to large, multicenter randomized controlled trials. Furthermore, the lack of quantitative evidence synthesis means that learning curve thresholds and cost figures reflect a range of reported values across heterogeneous studies rather than statistically derived pooled estimates, limiting the generalizability of the conclusions drawn. Future systematic reviews and meta-analyses incorporating formal quality assessment will be essential to build on the framework established here. Lastly, as mentioned previously, cross-study comparisons of monetary values should be interpreted with caution, as the cited studies vary significantly in many aspects and key methodological characteristics.

## 4. Conclusions

The evidence reviewed here demonstrates that minimally invasive surgery has fundamentally advanced the management of colorectal cancer. However, realizing the full potential of these platforms requires confronting two interconnected challenges that remain unresolved: the steep, multidimensional learning curves that demand substantial case volume and institutional commitment before consistent proficiency is achieved, and the considerable financial burden that continues to limit equitable access, particularly for robotic surgery. It is plausible and supported by indirect evidence that learning curves may have a direct impact on costs in this space; prolonged learning curves likely extend operative time, increase operating room and surgical instrumentation expenses, lengthen hospital stays, and lead to higher complication rates with their own financial burdens early in training. These observations underscore the need for strategies and data that simultaneously shorten learning curves, reduce overall costs, and promote equitable access to minimally invasive platforms. Defining more precise learning curve thresholds, reducing procedural costs, and ensuring that advanced minimally invasive techniques are available to all patients regardless of socioeconomic status must therefore be central priorities for the surgical community in the years ahead.

The following implementation strategies are proposed based on the evidence synthesized in this review and the authors’ clinical experience; where direct evidence is limited, these should be interpreted as informed recommendations requiring prospective validation rather than evidence-based directives. Translating the evidence into routine clinical practice requires targeted implementation strategies across educational, clinical, and institutional levels. For surgeons and training programs, the clear distinction between initial learning phases and technical mastery highlights that traditional case-volume targets alone are likely insufficient. Residency and fellowship curricula may benefit from adopting competency tracking, incorporating operative time, conversion rates, and lymph node yield, supported by structured simulation to safely accelerate early platform-specific learning curves. For healthcare institutions, balancing high upfront platform costs against downstream savings in hospital length of stay and lower complication rates and readmissions requires careful patient selection pathways. A potential institutional strategy that would require prospective evaluation includes prioritizing robotic platforms for anatomically challenging proctectomies, where technical advantages are most pronounced, while maintaining cost-effective laparoscopic pathways for theoretically less complex colon resections. However, executing this strategy effectively would benefit from close collaboration between hospital administrators and colorectal surgery teams based on individual surgeon competencies to reduce costs while maintaining excellent outcomes. Finally, surgical departments should consider implementing intraoperative cost-containment measures, such as standardizing instrument setups and optimizing operating room efficiency, to enhance the economic viability of these programs as robotic platforms gain broader adoption.

## 5. Future Directions

Several important issues remain to be addressed as minimally invasive colorectal surgery continues to evolve. First, there is a pressing need for standardization in how surgical learning curves are defined, measured, and reported. The considerable variability in proficiency thresholds across the existing literature is largely attributable to heterogeneity in analytic methods, outcome metrics, and case selection, underscoring the value of consensus guidelines. The latter could potentially be developed through a Delphi process or a collaborative multi-institutional study. This would allow us to establish uniform benchmarks for both laparoscopic and robotic colorectal procedures. The role of structured simulation curricula, proctorship models, and dedicated minimally invasive surgery fellowships in accelerating the learning curve and improving early-phase patient outcomes also warrants rigorous prospective evaluation, particularly as robotic platforms become increasingly central to colorectal surgical training programs.

In addition, addressing financial and equity issues relating to robotic colorectal surgery is another critical area for future investigation. The growing availability of alternative robotic platforms and reusable instrumentation offers a promising pathway toward reducing the upfront costs that currently limit broader adoption, and health economic research should evaluate the cost-effectiveness of these emerging systems relative to established platforms. Simultaneously, a space for policy reimbursement reform and value-based care models may be explored as mechanisms to bridge the access gap between patients with private insurance and those reliant on public payers or uninsured.

## Figures and Tables

**Figure 1:**
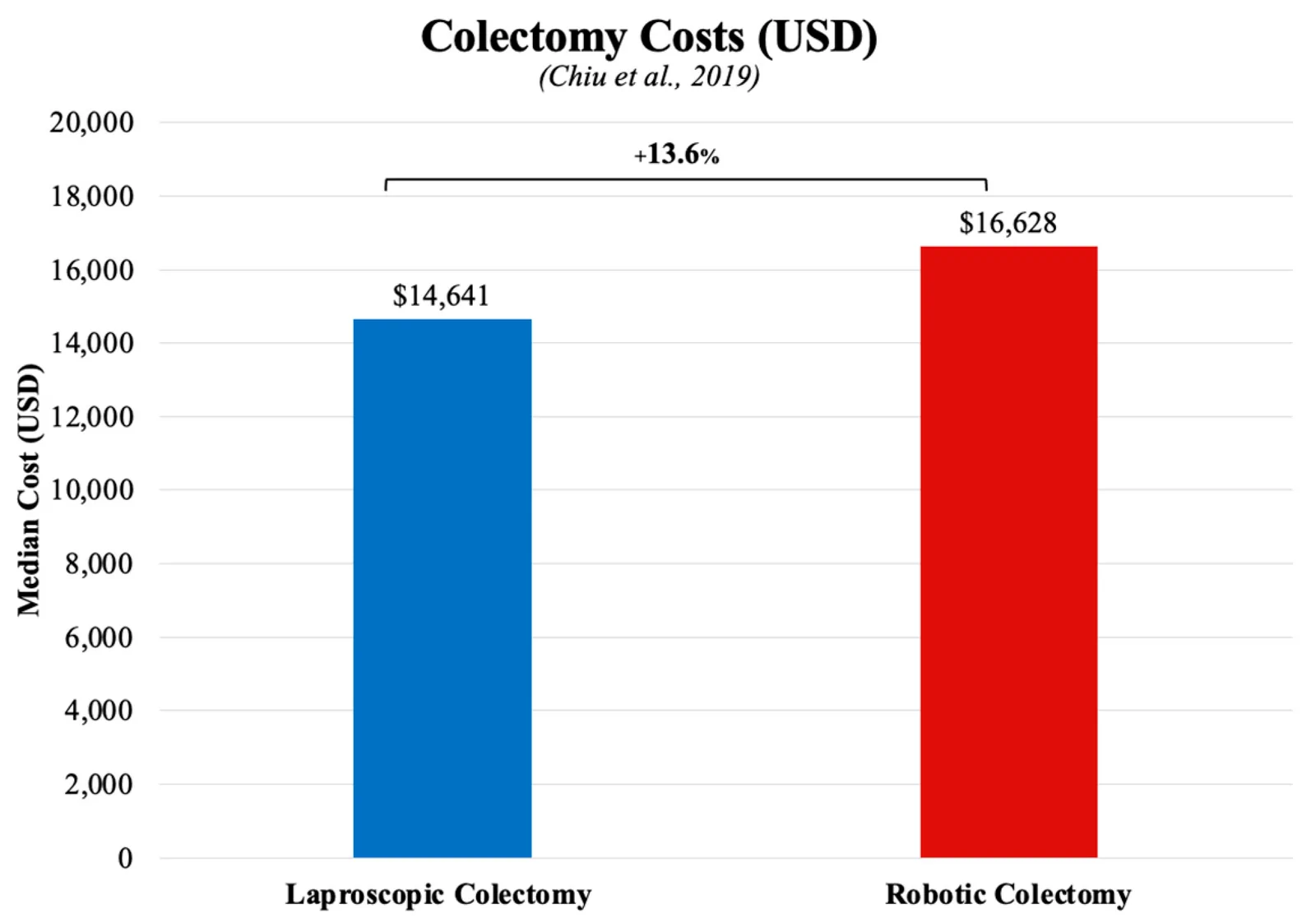

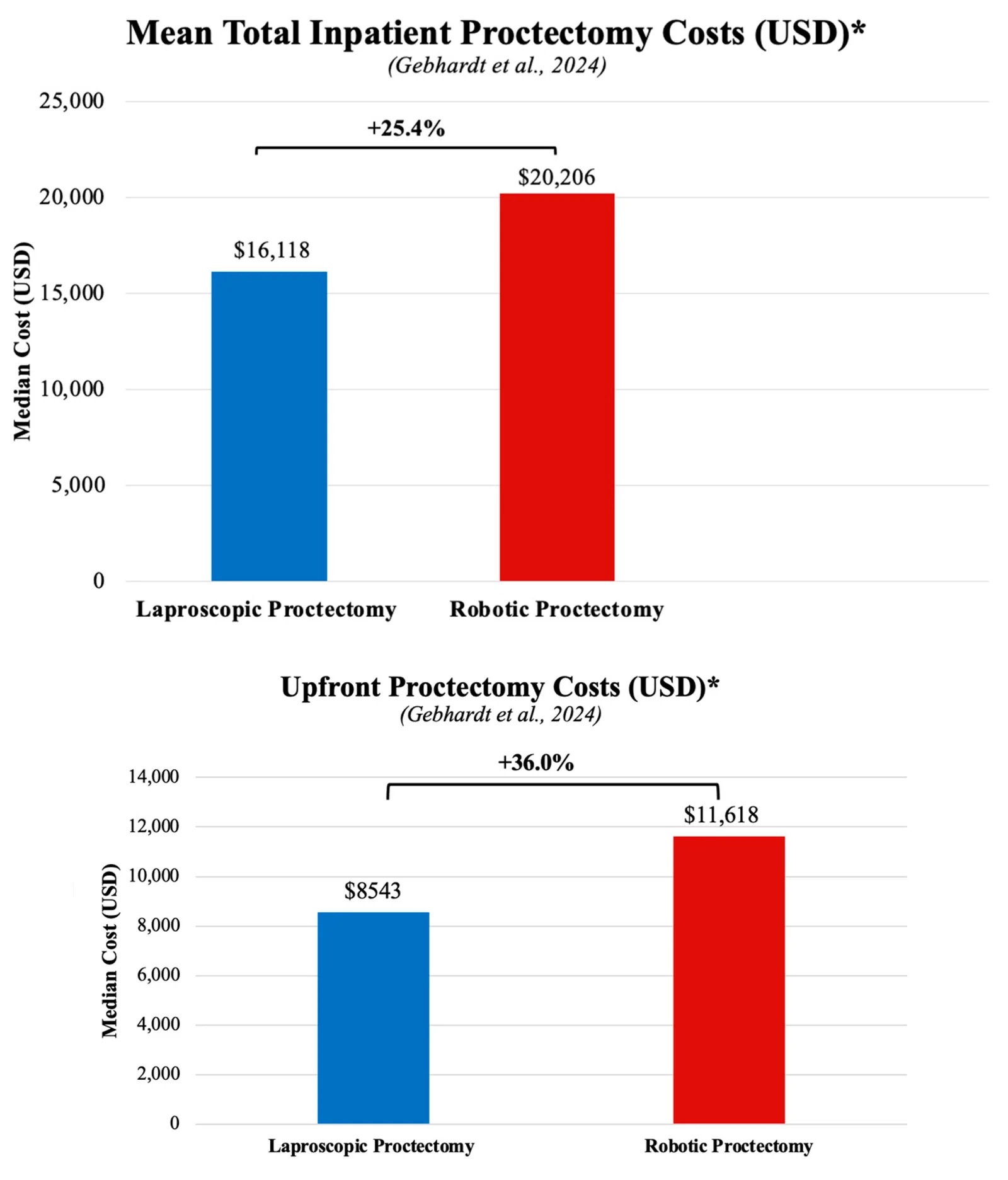
Cost Considerations for Robotic versus Laparoscopic Colectomies and Proctectomies. * Colectomy costs reported by Chiu et al. [32]. Median total inpatient costs for robotic and laparoscopic proctectomies were reported in euros by Gebhardt et al. [46] and converted to 2021 U.S. dollars using the mean EUR/USD exchange rate during the study period (2016–2021; 1 EUR = 1.144 USD). Converted units are rounded to the nearest US dollar. These converted values are intended to facilitate comparison with colectomy cost data and should be interpreted in the context of differing healthcare systems and study populations. It should be noted that the monetary comparisons demonstrated in this figure originate from different studies and healthcare settings, and hence do not represent a single direct economic comparison. The conversion of euro-denominated data to U.S. dollars does not account for inflation, differences in purchasing power, or health-system differences.

**Table 1:** Learning Curve Summaries for Robotic versus Laparoscopic Colectomies and Proctectomies.

Procedure Type	Study	Study Type	N (Patients)	Overall Learning Curve (Cases)	Operative Time Proficiency	Conversion Rate Proficiency (Cases)	Complication Proficiency (Cases)
Laparoscopic Colectomy	Tekkis et al. (2005) [9]	Retrospective cohort	900	55 (right), 62 (left)	Significant decrease with experience	—	—
Laparoscopic Colorectal Surgeries	Miskovic et al. (2012) [10]	Systematic review multicenter analysis, Surgeon Report	4852	—	96 cases	152	143
Laparoscopic Colectomy	Park et al. (2009) [11]	Retrospective cohort	1000	—	Continuous decrease (right hemicolectomy anterior resection)	—	—
Laparoscopic Colectomy	Giani et al. (2022) [12]	Prospective cohort	202	24–33	—	—	—
Robotic Colectomy	Parascandola et al. (2021) [14]	Retrospective cohort	502	None for right hemicolectomy; 35–40 cases for left hemicolectomy/sigmoidectomy	Significant reduction (left/sigmoid, 1st to 4th quartile); no significant change (right)	—	—
Robotic Colectomy	Parisi et al. (2017) [15]	Prospective cohort	108	44	44 cases	44	—
Robotic Colectomy	de’Angelis et al. (2016) [16]	Retrospective cohort (single fellow)	30	16	Significant decrease over time	—	—
Laparoscopic Proctectomy	Burghgraef et al. (2022) [17]	Systematic review	Avg 205 L-TME patients/study (45 studies)	50–70	—	—	—
Laparoscopic Proctectomy	Son et al. (2010) [18]	Retrospective cohort (single surgeon)	431	60–80	61–75 cases	61	79
Laparoscopic Proctectomy	Park et al. (2009) [11]	Retrospective cohort	1000	—	Decline after 90 cases	—	—
Laparoscopic Proctectomy	Bege et al. (2010) [19]	Retrospective cohort (single surgeon)	127	50	Gradual decrease over time	None	45
Laparoscopic Proctectomy	Kayano et al. (2011) [20]	Retrospective cohort	250	50	—	Decreased between 151 and 200	Decreased between 201 and 250
Laparoscopic Proctectomy	Kim et al. (2014) [21]	Retrospective cohort	317	110 cases (locoregional recurrence); 50–70 cases (CRM involvement)	—	—	—
Robotic Proctectomy	Burghgraef et al. (2022) [17]	Systematic review	Avg 150 R-TME patients/study (45 studies)	32–75	12–35	—	—
Robotic Proctectomy	Kim et al. (2014) [24]	Retrospective cohort (single surgeon)	167	32	Two peaks at 33 and 72 cases	—	—
Robotic Proctectomy	Tang et al. (2022) [25]	Retrospective cohort (single surgeon)	389	36	Steady decrease, especially in mastery phase	—	—
Robotic Proctectomy	Lee et al. (2021) [26]	Retrospective cohort (single surgeon, PSM)	506	Phase 1: 1–177; Phase 2: 178–506	—	—	—
Robotic Proctectomy	Arquillière et al. (2023) [27]	Prospective multicenter cohort	174	—	12–21	9–14	—

## Data Availability

No new data were created or analyzed in this study. Data sharing is not applicable to this article.
